# Utilizing Whole Genome Sequencing to Investigate a COVID-19 Cluster Among Healthcare Workers in a Tertiary Care Facility in the Philippines: Insights and Implications for Infection Prevention and Control

**DOI:** 10.1093/cid/ciaf057

**Published:** 2025-07-01

**Authors:** Gina de Guzman Betito, Reed Magleby, Janice C Caoili, Jason Caravas, Mark Lemuel Ybañez, Kara Moser, Matthew Westercamp, Maria Tarcela Gler, Global Action in Healthcare Network Healthcare-associated Infection Team, Morgane Donadel, Emily Petersen, Rachel Smith, Alle Taylor, Mardee Allen Balde, Jessica McLean, Maria Clarina Mariano, Fernanda Lessa, Matthew Westercamp, Reed Magleby, Morgane Donadel, Emily Petersen, Rachel Smith

**Affiliations:** Infection Prevention & Control Unit, Makati Medical Center, Makati, Philippines; Division of Healthcare Quality Promotion, National Center for Emerging and Zoonotic Infectious Diseases, US Centers for Disease Control and Prevention, Atlanta, Georgia, USA; Infection Prevention & Control Unit, Makati Medical Center, Makati, Philippines; Division of Infectious Disease Readiness and Innovation, National Center for Emerging and Zoonotic Infectious Diseases, US Centers for Disease Control and Prevention, Atlanta, Georgia, USA; Infection Prevention & Control Unit, Makati Medical Center, Makati, Philippines; Division of Healthcare Quality Promotion, National Center for Emerging and Zoonotic Infectious Diseases, US Centers for Disease Control and Prevention, Atlanta, Georgia, USA; Division of Healthcare Quality Promotion, National Center for Emerging and Zoonotic Infectious Diseases, US Centers for Disease Control and Prevention, Atlanta, Georgia, USA; Infection Prevention & Control Unit, Makati Medical Center, Makati, Philippines

**Keywords:** COVID-19, SARS-CoV-2, whole genome sequencing, phylogenetic analysis, cluster investigation

## Abstract

**Background:**

The coronavirus disease 2019 (COVID-19) pandemic has highlighted the importance of genomic surveillance and whole genome sequencing (WGS) for identifying mutations and supporting epidemiologic investigations. Healthcare workers (HCWs) face unique risks for COVID-19, potentially amplifying outbreaks within healthcare facilities (HCFs). This report details the use of WGS to retrospectively investigate a COVID-19 cluster among HCWs in a tertiary care HCF in the Philippines.

**Methods:**

Epidemiologic investigation was conducted by the HCF infection prevention and control (IPC) staff. The Global Action in Healthcare Network (GAIHN) COVID-19 variant characterization project retrospectively conducted WGS on selected HCW and inpatient respiratory specimens associated with the cluster with reverse-transcription polymerase chain reaction cycle threshold ≤32. Phylogenetic analyses were conducted using Nextstrain. Subclusters were defined by shared severe acute respiratory syndrome coronavirus 2 (SARS-CoV-2) lineage and epidemiologic data.

**Results:**

Investigation by IPC staff identified 19 HCWs with COVID-19 diagnosed during 2–9 September 2022 from a single nursing unit. Specimens for WGS were collected from 8 of these HCWs and from 43 additional HCF staff and inpatients with COVID-19 diagnosed from 1 August through 30 September 2022. Phylogenetic analyses identified 12 unique SARS-CoV-2 lineages and 2 subclusters: subcluster A (BA.5.2 lineage, n = 6) and subcluster B (BA.5.10.1 lineage, n = 7). Pairwise substitution-by-site analyses, combined with epidemiological data, provided support for multiple potential transmission events.

**Conclusions:**

WGS identified SARS-CoV-2 subclusters associated with high-risk exposure settings among HCWs in a tertiary care facility, providing essential insights into transmission pathways and demonstrating its potential to guide targeted IPC interventions and improve outbreak response strategies.

## Teaser

Whole genome sequencing (WGS) can improve understanding of SARS-CoV-2 transmission dynamics within healthcare facilities, providing valuable insights for infection prevention and control and suggesting additional value in enhancing WGS infrastructure, training, and collaboration for future outbreak responses.

The coronavirus disease 2019 (COVID-19) pandemic has demonstrated a role for genomic surveillance in informing public health action. Since its first characterization [1], whole genome sequencing (WGS) of severe acute respiratory syndrome coronavirus 2 (SARS-CoV-2) has been conducted to identify mutations and support epidemiologic investigations. Although the emergence of new variants is expected, and most have no known impact on viral behavior, some mutations may produce changes in phenotype. The public health risks of known and emerging SARS-CoV-2 variants include increased transmissibility, atypical clinical course, diagnostic failure, and decreased effectiveness of natural and vaccine-derived immunity [2].

Healthcare workers (HCWs) represent a population with unique risks for SARS-CoV-2 acquisition and transmission between HCWs and from HCWs to patients [3–5]. Due to their unique position at the interface between healthcare facilities (HCFs) and the community, HCWs can contribute to initiating or amplifying healthcare setting outbreaks, with significant implications for facility staffing and patient safety [6]. Although WGS has been used to support epidemiologic investigations and investigate healthcare transmission of SARS-CoV-2 [7–10], its use for these purposes has largely only been reported in high-income countries despite the ongoing global relevance of WGS to support detection of novel viral variants as endorsed by the World Health Organization [2, 11]. These studies have underscored the value of WGS in identifying lineage-specific transmission patterns that other approaches such as case investigation and contact tracing cannot. The Global Action in Healthcare Network (GAIHN) is a multinational network developed by the US Centers for Disease Control and Prevention (CDC) to address emerging infectious disease threats in healthcare settings. Since 2022, GAIHN has supported variant characterization of SARS-CoV-2 lineages affecting HCW and inpatients using WGS in HCFs in 4 middle-income countries: Brazil, Indonesia, Jordan, and the Philippines [12].

In the Philippines, the first suspected COVID-19 case was investigated on 22 January 2020 [13]. By September 2022, 64% of the country's total population had completed a primary series of a COVID-19 vaccine [12], with available vaccines including formulations from Sinovac, Pfizer-BioNTech, Moderna, and Oxford-AstraZeneca, among others. During September 2022, metropolitan Manila, where 90% of the population had completed a primary series of a COVID-19 vaccine, reported the highest number of cases in the country, with WGS surveillance indicating predominant circulation of BA.5, BA.4, BA.2.75, BA.5, BA.4, and BA.2.12.1 sublineages [14]. In parallel to the surge in community cases, a Manila tertiary care HCF participating in GAIHN observed a surge in COVID-19 cases, with 226 and 281 cases in August and September 2022, respectively. In this report, we describe the use of WGS to augment the investigation of a COVID-19 cluster among HCWs in a tertiary care HCF in the Philippines.

## MATERIALS AND METHODS

### SARS-CoV-2 Variant Characterization

The SARS-CoV-2 variant characterization project, as part of GAIHN, was a prospective, multisite, descriptive initiative characterizing circulating SARS-CoV-2 variants at the HCF level. This was achieved through secondary analysis of SARS-CoV-2–positive respiratory specimens collected from HCWs and inpatients with COVID-19 who were initially identified using facility procedures for SARS-CoV-2 diagnostic testing. Once confirmed as SARS-CoV-2 positive with reverse-transcription polymerase chain reaction (RT-PCR) testing, specimens underwent genetic characterization using sequencing-based methods. Specimens with a RT-PCR cycle threshold (Ct) value of ≤32 were deemed eligible for sequencing. To provide context for collected specimens, epidemiologic data, including patient demographics, information on symptoms, and vaccination history, were collected from medical record reviews and/or individual interviews. For HCWs, vaccination status was self-reported and verified by HCF vaccination records. For patients, only self-reported vaccination status was obtained. Verbal informed consent was obtained from all participants before conducting SARS-CoV-2 sequencing and collection of epidemiologic data.

### Infection Prevention and Control Investigation

Independent of GAIHN activities, the facility's infection prevention and control (IPC) team regularly reviewed positive SARS-CoV-2 results among HCWs and inpatients to identify clusters, utilizing standard epidemiologic criteria such as time, place, and individual characteristics. This approach was used to discern patterns and potential transmission dynamics within the facility, aiding in targeted containment efforts and further epidemiological investigations.

In September 2022, the facility IPC team investigated a potential outbreak among nurses in a surgical unit. HCWs from the surgical unit who tested positive by rapid antigen test from 2 September through 9 September 2022 were interviewed to collect information on symptoms, SARS-CoV-2 infection history, vaccination status, and potential exposures.

### Selection of HCF Samples for Genomic Analysis

We used the existing GAIHN SARS-CoV-2 surveillance methodology and supplemented it with additional genomic analyses to support the IPC investigation. HCF cases included as part of the genomic analyses were either (*i*) HCWs from the suspected surgical unit cluster; (*ii*) additional HCWs from the surgical unit with symptom onset dates from August 3 through 18 September 2022, but who were not initially identified as part of the surgical unit case cluster; (*iii*) HCWs from outside the surgical unit with symptom onset dates from 3 August through 18 September 2022; or (*iv*) hospitalized patients with symptom onset dates from 3 August through 18 September 2022. All specimens underwent confirmatory testing by RT-PCR. Verbal informed consent for genomic analysis with WGS was obtained for those who tested positive by RT-PCR confirmatory testing.

### SARS-CoV-2 WGS and Variant Characterization

Specimens eligible for sequencing were purified and underwent RNA extraction, followed by sequencing on the Miseq v3 platform at the Philippine Genome Center. Library preparation was performed using the Illumina COVIDseq protocol with v3 primers, and library quality was assessed with Tapestation 2200 with quantification using Qubit fluorometer. SARS-CoV-2 lineages were assigned using Pangolin nomenclature [15], and whole genome assemblies were reported to the facility. All WGS results were uploaded to the Global Initiative on Sharing All Influenza Data (GISAID).

### Subcluster Definition

To further characterize the HCF cases, we defined subclusters using combined spatiotemporal and genomic data. A subcluster was defined as individuals with a shared epidemiologic link within the surgical nursing unit, symptom onset dates within a 14-day period, and respiratory specimens from the same variant Pangolin lineage.

### Phylogenetic Analysis

Following the initial report of WGS results to the facility, we conducted additional phylogenetic analyses, supplementing our findings with additional publicly available data. To provide targeted and contextual data for the analysis of the HCF data, we supplemented the HCF dataset with 2 additional datasets from publicly accessible genomic databases: (*i*) 50 sequences sampled evenly over time from all regions globally that were reported prior to 20 November 2022, from the Nextstrain Open Data (GenBank) “Global” dataset; and (*ii*) 50 additional sequences collected in the Philippines during 12 May through 20 November 2022 sampled from a GISAID dataset of Philippine sequences (EPI_SET_230502fg; Supplementary Table 1) to represent the period 2 months prior, during, and 2 months after the HCF cluster investigation [16]. Phylogenetic analyses were performed using Nextstrain, and visualization of the phylogenetic tree was generated using Auspice 2.51.0 [17].

We conducted pairwise comparisons to further contextualize transmission for the 2 most prevalent lineages used to define our subclusters and, thus, those with the highest likelihood of identifying trends in genetic similarity. We generated consensus trees for both lineages from 1000 bootstrap replicates using iqtree v2.2.2.7. Using the Seaborn python package, we used this tree to generate a maximum likelihood–corrected distance matrix representing substitutions per site displayed as a heatmap [18].

## RESULTS

### Epidemiology of the IPC Investigation

From 2 to 9 September 2022, the IPC investigation identified 19 staff members who tested positive by SARS-CoV-2 rapid antigen testing as part of the surgical unit cluster. Of these 19 HCWs, 14 were able to be interviewed as part of the initial epidemiologic investigation.

Among those interviewed, all 14 presented with at least 1 symptom consistent with COVID-19, including fever or chills (10/14 [71%]), cough (13/14 [93%]), sore throat (10/14 [71%]), headache (8/14 [57%]), muscle pain (8/14 [57%]), diarrhea (3/14 [21%]), and loss of taste or smell (1/14 [7%]). Symptom onset ranged from 30 August through 6 September 2022.

Ten cases (10/14 [71%]) reported a known exposure at work, while 4 (4/14 [29%]) had no known exposures, and 1 reported an exposure outside of work (1/14 [7%]). The majority of cases (8/14 [57%]) were nurses, 4 (4/14 [29%]) were physicians, and 2 (2/14 [14%]) were nonclinical staff. All participants had received at least 2 doses of a COVID-19 vaccine.

Among the 14 cases in the surgical unit cluster who were interviewed as part of the epidemiologic investigation, 12 agreed to participate in the genomic analysis as part of the GAIHN sequencing initiative; 8 of these (8/12 [67%]) had respiratory specimens eligible for sequencing.

### Genomic Analysis

**Figure 1.**
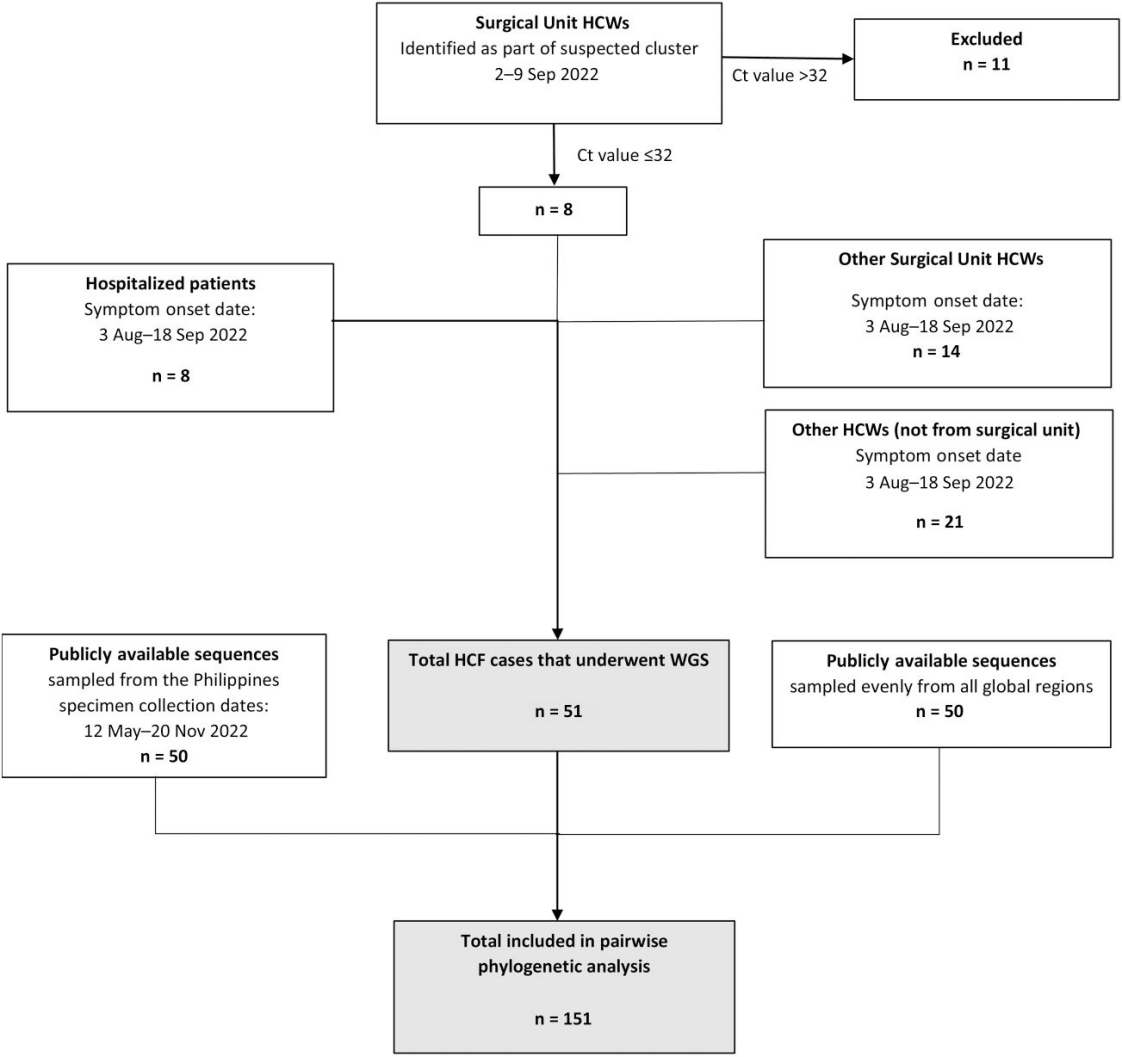
Flow diagram for selection of samples for genomic analysis including those from the study healthcare facility in the Philippines and from publicly available sequences, May–November 2022. All healthcare worker specimens included had a cycle threshold value ≤32. Abbreviations: Ct, cycle threshold; HCF, healthcare facility; HCW, healthcare worker; WGS, whole genome sequencing.

To supplement the investigation, an additional 43 specimens from the same HCF facility collected as part of the GAIHN sequencing initiative, with symptom onset dates from 3 August through 18 September 2022, were also included in the genomic analysis, providing a combined total of 51 unique specimens that underwent WGS. Among the 51 were 8 HCWs from the initial cluster investigation with respiratory specimens eligible for sequencing, 14 HCWs from the surgical unit who were not considered part of the original cluster investigation, 21 HCWs from outside of the surgical unit, and 8 hospitalized patients. A flow diagram for the selection of cases for genomic analysis is shown in Figure 1.

Demographics, symptoms, and WGS results of all 51 HCF participants are summarized in Table 1. Almost all HCF participants included in the genomic analysis (49/51 [96%]) were symptomatic, and almost all (50/51 (98%) reported that they had received at least 2 COVID-19 vaccine doses. Most HCF participants (43/51 [84%]) were HCWs, but fewer than half (22/51 [43%]) had a link to the surgical unit cluster. The average N gene Ct value was 21.5 (range, 13.3–31.2), and most specimens (44/51 [86%]) had an N gene Ct value between 15 and 30 (Table 1). Among the 8 admitted patients included in the analysis, admission dates ranged from 16 July to 7 September 2022. The median time between admission and symptom onset date was −0.5 days (ie, symptom onset date 0.5 days before admission), with a range of −36 days to 10 days.

**Table 1.** Summary of Basic Demographic and Pangolin Lineages Identified Among Participants Included in the Study From a Single Healthcare Facility in the Philippines, August–September 2022

Characteristic	All Participants (n = 51)		Subcluster A (n = 6)		Subcluster B (n = 7)	
Healthcare worker	43	(84%)	5	(83%)	7	(100%)
At least 2 vaccine doses	50	(98%)	6	(100%)	7	(100%)
Symptomatic	49	(96%)	6	(100%)	7	(100%)
Reported exposure at HCF	21	(41%)	1	(17%)	7	(100%)
Link to cluster surgical unit	22	(43%)	5	(83%)	7	(100%)
Specimen collection date						
July 2022	2	(4%)	0	…	0	…
August 2022	24	(47%)	6	(100%)	1	(14%)
September 2022	25	(49%)	0	…	6	(86%)
Ct value (N gene)						
<15	5	(10%)	1	(16%)	0	…
15–30	44	(86%)	4	(67%)	6	(86%)
>30	2	(4%)	1	(17%)	1	(14%)
SARS-CoV-2 lineage						
BA.2	1	(2%)	0	…	0	…
BA.2.3.20	8	(16%)	0	…	0	…
BA.4.1	1	(2%)	0	…	0	…
BA.5.10.1	12	(24%)	0	…	7	(100%)
BA.5.2	15	(29%)	6	(100%)	0	…
BA.5.2.1	3	(6%)	0	…	0	…
BA.5.2.20	1	(2%)	0	…	0	…
BA.5.2.62	2	(4%)	0	…	0	…
CM.2	3	(6%)	0	…	0	…
CM.4	2	(4%)	0	…	0	…
CM.5	1	(2%)	0	…	0	…
CM.5.1	1	(2%)	0	…	0	…
CM.5.2	1	(2%)	0	…	0	…

Data are presented as No. (%).

Abbreviations: Ct, cycle threshold; HCF, healthcare facility; SARS-CoV-2, severe acute respiratory syndrome coronavirus 2.

Using spatiotemporal and genomic data, we identified 2 distinct subclusters among the HCF participants included in the genomic analysis. One subcluster (subcluster A, n = 6) was associated with the BA.5.2 lineage, and the other (subcluster B, n = 7) was associated with the BA.5.10.1 lineage (Supplementary Figure 1).

**Figure 2.**
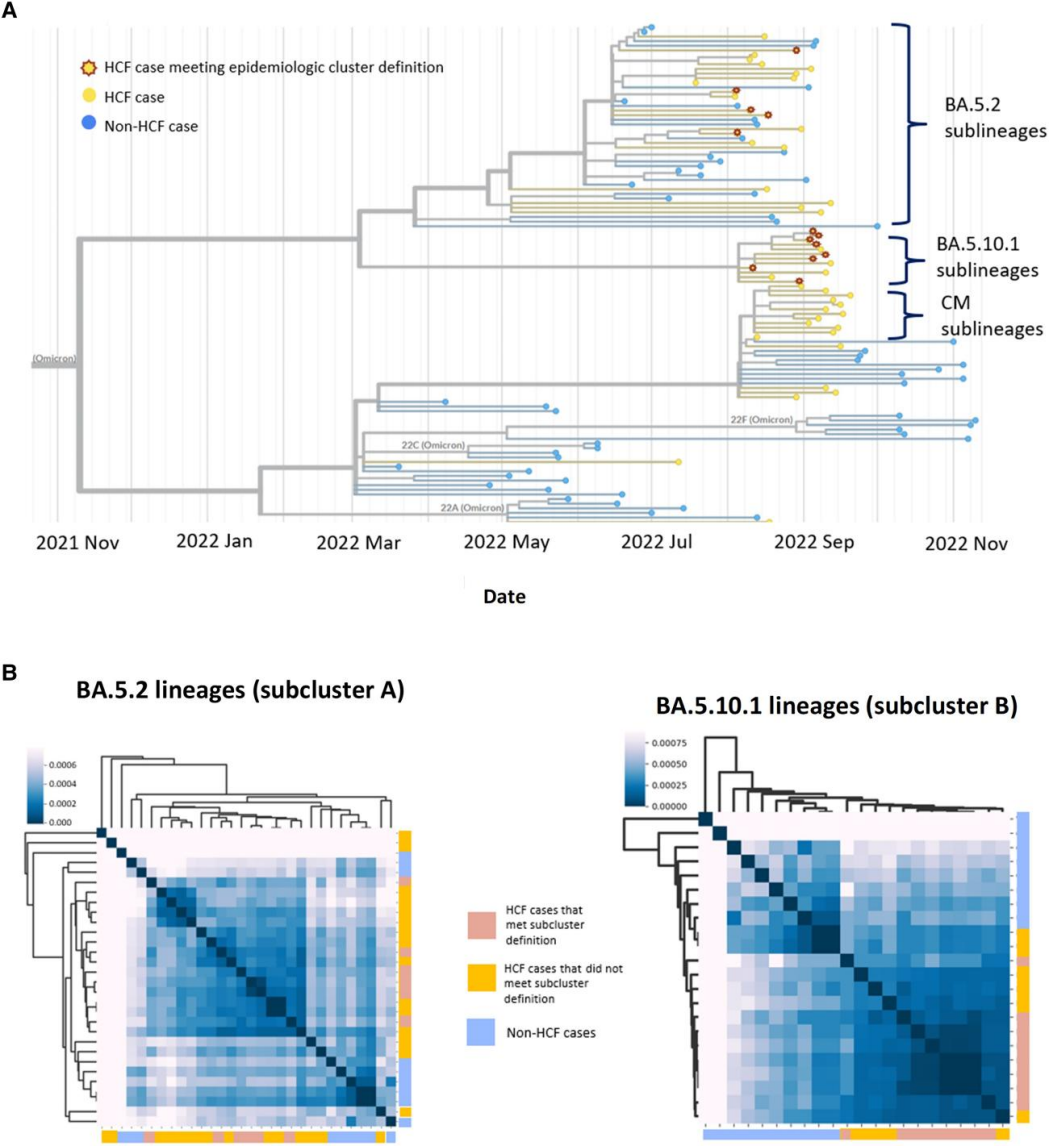
*A*, Phylogenetic tree of whole genome sequences isolated from (*i*) Global Action in Healthcare Network (GAIHN) study healthcare facility (HCF) cases, August–September 2022; and (*ii*) other Philippine cases identified from the Global Initiative on Sharing All Influenza Data (GISAID), March–November 2022. For readability, only 56 non-HCF sequences are shown, selected semi-randomly from GISAID data by prioritizing even temporal sampling across March–November 2022. An untrimmed tree is included in the Supplementary Material. *B*, Heatmaps of healthcare facility sequences compared to publicly available non-HCF sequences for specimens identified as BA.5.10.1 and BA.5.2 lineages. Shading represents maximum likelihood–corrected pairwise genetic similarity defined by substitutions per site.

The turnaround time from initial cluster identification to WGS results available to the facility was 33 days, and preliminary findings from phylogenetic analyses were shared with the facility 68 days after the initial identification of the potential surgical unit cluster. The phylogenetic tree, shown in Figure 2*A*, incorporates data from the HCF and publicly available sequencing data from the Philippines from 12 May to 20 November 2022. Sublineages of BA.5.2 (BA.5.2, BA.5.2.1, BA.5.2.20, BA.5.2.62; 21/51 [41%]), BA.5.10.1 (12/51 [24%]), and CM (CM.2, CM.4, CM.5, CM.5.1, CM.5.2; 8/51 [16%]) accounted for the majority of analyzed specimens from the HCF. Pairwise substitution-by-site analyses for the 5.10.1 sublineages showed a subset of HCF cases within the subcluster with high genetic relatedness compared to non-subcluster HCF and non-HCF cases.

### Characteristics of Subcluster Patients

All individuals in subcluster A reported 1 or more symptoms (6/6 [100%]), with symptom onset dates ranging from 4 to 24 August 2022. Most were HCWs within the same surgical nursing unit (5/6 [83%]; 3 physicians and 2 nurses), all of whom were involved in patient care (Table 1). Only 1 individual had reported a known exposure to a confirmed or suspected COVID-19 case in the facility; 2 had no known exposures, and 2 had a known household exposure. Of the 5 HCWs, 1 required HCF admission for treatment. No deaths occurred.

All individuals in subcluster B were symptomatic, with symptom onset dates ranging from 22 August to 5 September 2022. All (7/7 [100%]) were HCWs within the same surgical nursing unit (4 physicians, 3 nurses), all of whom were involved in patient care. All reported an exposure in the facility. None required admission for treatment, and no deaths occurred (Table 1).

Four (4/7 [57%]) HCWs within subcluster B, along with 1 HCW not identified as part of the original surgical unit cluster identified by the IPC investigation, were found to have evidence of genetic similarity based on maximum likelihood–corrected genetic distance (Figure 2*B*). The HCW who was found to have evidence of genetic similarity but did not meet our subcluster cluster definition was a nurse who worked in a nursing unit caring for patients with confirmed or suspected COVID-19 but who denied any known exposures in the community or in the HCF without the use of appropriate personal protective equipment. A follow-up interview performed after the analysis results were available did not identify specific epidemiologic links with known cases from operative services.

## DISCUSSION

This report provides a retrospective description of a cluster of HCWs and inpatients with COVID-19 at a tertiary care HCF in the Philippines, using WGS results from data collected as part of an ongoing surveillance activity. The genomic analyses supplemented the initial HCF cluster investigation by identifying subclusters based on SARS-CoV-2 lineages, providing evidence of possible transmission within the facility.

Although WGS is performed throughout the Philippines, results are rarely shared with clinical staff for situational awareness and IPC consideration, and metadata are often not linked to these specimens. In contrast, the results of these analyses were presented to facility IPC leadership and staff, thereby allowing for additional contextualization of SARS-CoV-2 transmission dynamics in their facility. The findings supported the original conclusion of the facility-led cluster investigation, suggesting that infection transmission among staff occurred. Our WGS results strengthened these conclusions with evidence of transmission supported by pairwise genetic comparisons. Additionally, pairwise comparisons within sublineage groups helped control for potential temporal shifts in sublineages circulating in the community. Although the retrospective nature of the study did not allow for use of the results to avert further transmission, the additional context provided by the WGS results and phylogenetic analyses allowed for a reevaluation of existing IPC protocols to inform response planning at the facility and raise awareness around potential SARS-CoV-2 transmission within the HCF and between HCWs, and provided a well-described and evidence-supported case study for future staff response and infection prevention training.

The WGS activities were supported by the CDC as part of GAIHN. However, resource limitations have been highlighted as an important barrier to the implementation of WGS activities in HCFs globally [11] and contribute to the paucity of reported results from WGS activities in low- and middle-income countries (LMICs). Specimens were collected from a highly vaccinated population during a period in which this region of the Philippines was seeing the highest burden of cases in the country [14].

This facility was the first in the Philippines to conduct SARS-CoV-2 WGS activities as part of GAIHN. As expected, given the design of the surveillance initiative, the 33-day turnaround time limited the ability to use the genomic findings for immediate outbreak response. While the benefits of rapid turnaround times for WGS to inform outbreak response are well established [19], the World Health Organization recommends balancing the integration of WGS into existing HCF laboratory operations with potential disruptions and limitations related to staffing, training, and funding [20–22].

This analysis had several limitations. The GAIHN variant genomic surveillance initiative is designed as a passive system relying on facility procedures for case identification and initial diagnostic testing. Therefore, the results presented may underestimate the extent of this case cluster due to failure to identify possible cases, in-facility testing not being performed, or individuals refusing inclusion in the surveillance initiative. The passive design also limited our ability to prioritize specimen collection as soon as possible after symptom presentation, which may have increased the Ct values of collected specimens, thereby affecting the genetic results. Limitations in genetic results were partially addressed by incorporating epidemiologic criteria in our subcluster definition and using a pairwise substitution-per-site analysis that did not rely solely on the quantification of single-nucleotide polymorphisms.

This retrospective analysis underscores the value of WGS in understanding SARS-CoV-2 transmission within HCFs. By integrating WGS with epidemiologic data, additional cases and transmission pathways were identified, enhancing the conclusions of investigations conducted by the IPC team and informing prevention efforts. Additionally, these findings support conclusions from prior studies which suggest that WGS may be able to identify transmission events that are undetected by epidemiological analysis [2]. Although resource-intensive, WGS can provide additional tools to augment investigations of SARS-CoV-2 clusters by HCF IPC staff and better understand transmission. Findings from WGS can potentially help guide IPC interventions in real time, for example by prioritizing high-risk populations for enhanced surveillance, adjusting staff workflows, and identifying high-risk areas for disinfection. To optimize the use of WGS, HCFs should consider establishing systems that address the specific requirements of genetic methods in specimen collection, processing, informatics, and communication of results. When resources for WGS are limited, HCFs should additionally identify opportunities for collaboration with regional WGS partners and leverage existing public health infrastructure, such as reference laboratories, where available. Investing today in staff training, building the necessary infrastructure incrementally, and fostering collaboration with reference laboratories will improve the timeliness and value of WGS in the future. Future research priorities should focus on methods to reduce WGS turnaround time and improve understanding of the cost-effectiveness of WGS of viral pathogens in low-resource settings. Addressing these priorities and operational challenges, especially in LMICs, will better equip us to respond to future outbreaks and protect HCWs and patients.

## Supplementary Material

ciaf057_Supplementary_Data

## Supplementary Data

Supplementary materials are available at *Clinical Infectious Diseases* online. Consisting of data provided by the authors to benefit the reader, the posted materials are not copyedited and are the sole responsibility of the authors, so questions or comments should be addressed to the corresponding author.

## Notes

***Acknowledgments.*** We recognize the contributions of other members of the GAIHN Healthcare-associated Infection team (Morgane Donadel, Emily Petersen, and Rachel Smith) and the assistance of Health Security Partners members Alle Taylor, Mardee Allen Balde, and Jessica McLean. Additionally, we are grateful to Maria Clarina Mariano for contributions to the initial outbreak investigation and the staff of the Philippine Genome Center for their technical contributions to this work. Finally, we gratefully acknowledge all data contributors to publicly available genomic datasets, that is, the authors and their originating laboratories responsible for obtaining the specimens, and their submitting laboratories for generating the genetic sequence and metadata and sharing via the Global Initiative on Sharing All Influenza Data (GISAID), on which part of this research is based. GAIHN Healthcare-associated Infection CDC team members: Fernanda Lessa, Matthew Westercamp, Reed Magleby, Morgane Donadel, Emily Petersen, Rachel Smith.

***Study approval.*** This study was approved by the CDC’s human subjects review committee and the ethics review committee at the healthcare facility.

***Financial support.*** This work was funded by the Global Action in Healthcare Network (GAIHN) developed by the US Centers for Disease Control and Prevention (CDC) through cooperative agreement number CK21-2104.
