# Supplementary material for: Utilizing Whole Genome Sequencing to Investigate a COVID-19 Cluster Among Healthcare Workers in a Tertiary Care Facility in the Philippines: Insights and Implications for Infection Prevention and Control
Source: Clin Infect Dis. 2025 Jul 1;80(6):1262–8. doi: 10.1093/cid/ciaf057 (PMC12272845; doi:10.1093/cid/ciaf057)
Supplement: ciaf057_Supplementary_Data [file ciaf057_supplementary_data.zip › CID-125254 Suppl Table.docx]

**Supplementary Table 1**

**Data Availability**

GISAID Identifier: EPI_SET_230502fg
DOI: 10.55876/gis8.230502fg

All genome sequences and associated metadata in this dataset are published in GISAID’s EpiCoV database. To view the contributors of each individual sequence with details such as accession number, Virus name, Collection date, Originating Lab and Submitting Lab and the list of Authors, visit https://doi.org/10.55876/gis8.230502fg

**Data Snapshot**

- Data Snapshot EPI_SET_230502fg is composed of 4,768 individual genome sequences.
- The collection dates range from 2022-05-14 to 2022-11-14;
- Data were collected in 1 country and territory; All sequences in this dataset are compared relative to hCoV-19/Wuhan/WIV04/2019 (WIV04), the official reference sequence employed by GISAID (EPI_ISL_402124).
