# Supplementary figures and images for: Utilizing Whole Genome Sequencing to Investigate a COVID-19 Cluster Among Healthcare Workers in a Tertiary Care Facility in the Philippines: Insights and Implications for Infection Prevention and Control
Source: Clin Infect Dis. 2025 Jul 1;80(6):1262–8. doi: 10.1093/cid/ciaf057 (PMC12272845; doi:10.1093/cid/ciaf057)

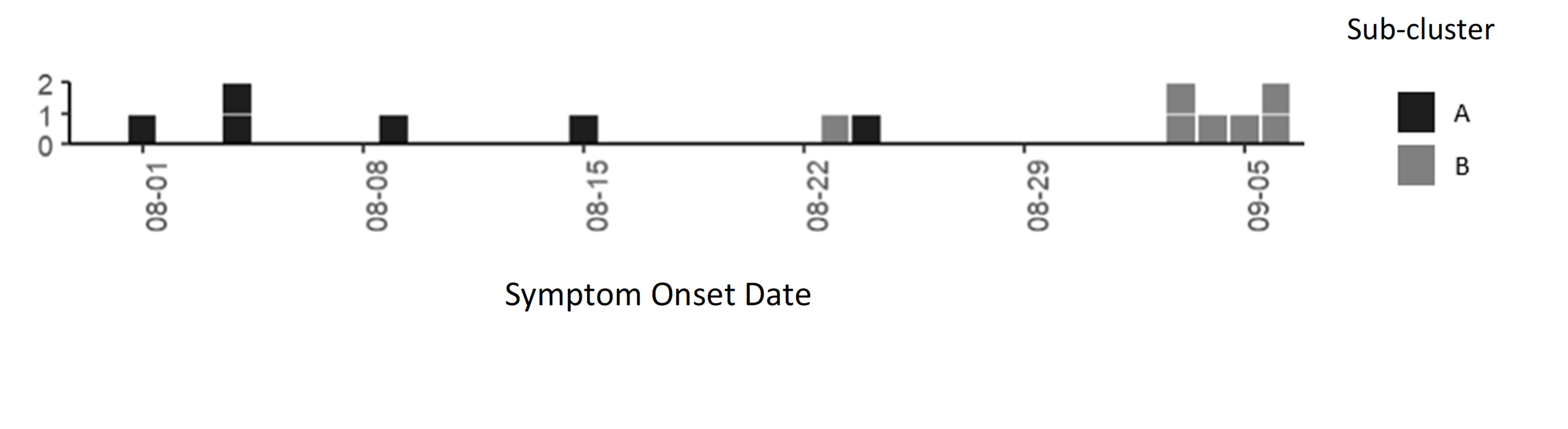

Supplement: ciaf057_Supplementary_Data [file ciaf057_supplementary_data.zip › SupplementaryFigure1.tif]

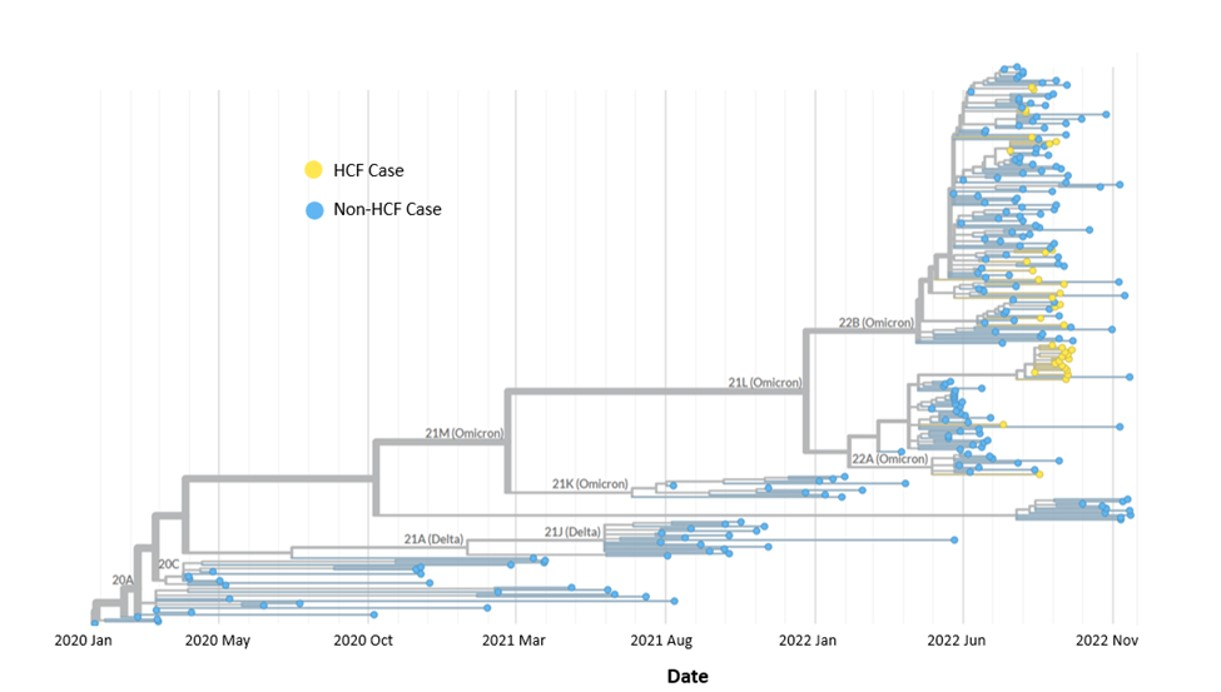

Supplement: ciaf057_Supplementary_Data [file ciaf057_supplementary_data.zip › SupplementaryFigure2_Magleby.tif]
